# Internet interventions and therapies for addressing the negative impact of digital overuse: a focus on digital free tourism and economic sustainability

**DOI:** 10.1186/s12889-023-17584-6

**Published:** 2024-01-13

**Authors:** Juan F. Arenas-Escaso, José A. Folgado-Fernández, Pedro R. Palos-Sánchez

**Affiliations:** 1 Department of Financial Economics and Accounting, University of Extremadura, Cáceres, Spain; 2 Department of Financial Economics and Operations Management, University of Sevilla, Sevilla, Spain

**Keywords:** Economic sustainability, Digital overuse therapies, Tourism management, Health and economy, Digital free tourism, Information technology, Promotion of health

## Abstract

**Background:**

The excessive use of information technologies (IT) and online digital devices are causing symptoms of burnout, anxiety, stress and dependency that affect the physical and mental health of our society, extending to leisure time and work relationships. Digital free tourism (DFT) is a phenomenon that emerges as a solution to technostress and pathologies derived from digital hyperconnection. The objective of this research is to advance the knowledge of new structures of motivational factors that can understand the decision of a tourist to make a DFT trip. To this end, it is investigated whether family and social engagement and health and relaxation have a positive impact on the behavioral intention of the potential tourist and whether this influences sustainability due to the importance of DFT in the new economic framework.

**Methods:**

With a quantitative approach, the methodology used consisted of an online questionnaire among potential travelers. IBM SPSS Statistics 22.0 statistical software was used to evaluate the data obtained and confirm the relationships of the model and the research hypotheses.

**Results:**

The results of the questionnaire assessed the contribution of each construct to the tourist’s behavioral intention and the tourist’s decision to make the decision to undertake a DFT experience.

**Conclusions:**

DFT can be a driver of economic sustainability and health therapy in tourism in the digital age. This study aims to expand the lines of research on DFT and determine the complex factors that can lead a tourist to participate in the DFT experience. The results obtained can help managers of companies in the sector to offer more efficient and sustainable services that contribute to the health and wellbeing of tourists as a differentiating factor.

**Supplementary Information:**

The online version contains supplementary material available at 10.1186/s12889-023-17584-6.

## Introduction

Currently, 5.32 billion people in the world use a smartphone, and 4 out of 5 mobile devices are active and permanently connected to the Internet [1]. In addition, three-quarters of the planet’s inhabitants use social networks as communication channels and for social interaction [2–4].

Numerous studies have demonstrated the usefulness of new technologies and social networks in information exchange, such as socialisation [5], mental health [6], improved self-esteem [7], emotional benefit [4], self-expression and increased quality of life for individuals [8, 9]. New technologies and social networks have enabled the improvement of living standards and changes in people’s consumption concepts have greatly boosted the development of tourism [10, 11], which has further benefited the hospitality sector [12, 13].

An increasing number of studies are addressing the need to limit the use of IT and reduce digital hyperconnection [3, 14, 15].

In recent years, these lines of research have focused on proposing therapies to combat the adverse health and wellbeing effects of this technological addiction [13, 16].

The research on addiction to the Internet and social media stands out in the scientific literature [11, 17] analyse the mechanical attitude and behaviour of users that lack self-control and self-awareness [18].

Other studies have revealed the negative impacts of IT on society, such as the influence of fake news [9], polarization of public opinion [19], data protection and privacy,especially in the health sector where patient data is highly sensitive and there are concerns that anonymisation of data is not sufficient to preserve patient privacy, cybercrime, addiction to being connected [20], the obsessive attraction of social media [21], hyperconnection of cyber workers [22], control of big data and virtual monetary systems without financial regulation [12, 23] or the new domain of Artificial Intelligence (AI) [24] and use of virtual reality [2].

The tourism sector has responded to the growing demand for digital disconnection trips and holidays by offering DFT experiences [25].

Digital Free Tourism (DFT) has become an attractive tourism market and an emerging business opportunity. Existing lines of research have studied the application of new technologies in business and the hospitality sector but have not considered the concept of digital disconnection and wellbeing in a holiday context [15, 17, 26].

In the field of tourism, the motivations of tourists on a DFT trip have been examined the effects that a DFT experience can have on well-being [7, 27–29] and also on health [9, 16, 30].

The results have identified DFT-derived attributes that provide important findings that can inform strategies in the tourism sector and its promotion as an emerging and future market [11, 31–33].

This phenomenon emerged in 2013 in the United States and extended throughout the world in only a few years, becoming a global emerging market opportunity for the tourism sector, wellness and health and for economic sustainability [4, 34–36].

DFT accommodation and travel agencies offer services for technological disconnection that limit access to information with alternative activities, exclusive stays free of electronic devices or therapies such as yoga, hiking, mindfulness and pilates, which offer to improve the well-being of the customers [15, 37].

There are strategies that try to help users temporarily disassociate from their digital devices or use them in a balanced and responsible way [38–40].

However, there are barriers to the decision to take a DFT travel. There a few studies on the behavioural intention of tourists to use experiences that limit the use of smartphones [3, 41].

This study addresses a new problem with a strong impact of technology and tourism. The methodology is based on an exploratory analysis, building on previous studies, using a survey of potential Spanish tourists. The scientific production of studies on Digital Detox and specific studies on Digital Free Tourism is very scarce. The use of structural equations to evaluate the results of the questionnaires is a novelty in the work as it employs a pioneering statistical analysis carried out with PLS-SEM and DFT [42].

The aim of this study is to examine the opportunities that DFT can bring in the tourism sector for tourism service providers and, in turn, to investigate the influence of tourists’ behavioural intentions (BI) on the variables offered by DFT attributes linked to social and family engagement, relaxation and wellbeing and connection with nature. It also studies the impact of BI on DFT economic sustainability in the new economic scenario and the complex relationship between digital technologies and tourism.

In summary, we use a quantitative approach that investigates the attitudes and motivations of potential DFT tourists by employing a new dimension, sustainability as a cornerstone of DFT attributes and its influence on the behavioural intention of these potential tourists.

**Table 1:** Terminology in digital free tourism travel and digital disconnection travel

Terminology DFT	Authors	Year	Topics
Digital detox holidays (DDH)	Jiang & Balaji	2022	Disconected in travels and holidays
	Winke	2010	Addiction and holidays
Digital detox tourism (DDT)	Gaafar	2021	Attitudes and motivators in Egyptian tourists
	Hoving	2017	Motivations Dutch tourists’ digital detox
	Pawloska-Legwad & Matoga	2020	Disconnect from digital world
	Wilckonson	2019	Effects smartphone: anxiety and craving
Digital free holidays (DFH)	Emek	2014	Addiction and digital free holidays
	Ozdemir	2021	Bibliometrics analysis about digital holidays
Digital free tourism (DFT)	Arenas et al.	2022	Opportunities DFT
	Dickinson et al.	2016	Disconnection at campsite
	Egger et al.	2020	Exploratory study DFT motivations
	Floros et al.	2019	Millennials
	Fryman & William	2021	Smartphones dependency
	Hassan et al.	2022	DFT Tourism and Well Being
	Li et al.	2018	Critical discourse digital free tourism
	Li et al.	2020	Character Strengths digital free tourist
	Liu & Hu	2021	Technostress perspective in digital free tourism
	Cai et al.	2020	Turn it off in travels
	Cai et al.	2023	Power and resistance in a connected world
Tourism offline	Syvertsen	2022	No access internet mountain. Experiences.
Unplugged in experiences. Motivations, attitudes	Ayeh	2018	Problematic use technology in holidays
	Durán-Román et al.	2021	Sustainability and experience at destination
	Kirillova & Wang	2020	Smartphones disconnected in holidays
	Kuntsman & Miyake	2015	Digital disengagement
	Paris et al.	2015	Campsites and disconnection
	Fan et al.	2019	Face to face contact in destination immersion
	Thomas et al.	2016	Benefits connection and disconnection
	Benedict et al.	2019	Benefits connection and disconnection
	Zhuang et al.	2021	Tourism experiences of AR technology use

Own authors

This research offers a thoughtful perspective to understand how providers can leverage DFT strategies to achieve greater appeal to potential travellers.

The drive for new technologies and digitalisation has led to the need to rethink business models and segments oriented towards the sustainability and viability of tourism resources. In this sense, service providers are making efforts to turn their destinations into service providers are making efforts to turn their destinations into ideal destinations that meet the needs and experiences of their potential customers [12, 17].

With these premises, tourism managers are starting to promote sustainable strategies in line with their clients’ offer. Environmental and economic sustainability is a priority objective for the new manifestations of tourism, such as DFT, which proposes and promotes the revaluation of authentic resources [43, 44]. For these reasons, research population would be clients of this promote sustainable strategies and with issues related with DETOX.

The research questions are as follows:

1. Investigate whether in the new digital era DFT can offer a competitive advantage in attracting tourists and be a driver of economic sustainability.
2. To identify DFT as a new business model and generator of business initiatives that promote health and wellness tourism.
3. Expand the field of knowledge of DFT by adding economic sustainability as a factor influencing the behavioural intention of tourists when seeking DFT experiences.

After this introduction, the existing scientific literature about DFT is reviewed. Then, the methodology of the research and the data collection system using an online questionnaire of 426 tourists are given, the results obtained are discussed, and the conclusions of the study are presented.

## Background

### Social and family engagement

Every individual wants to have social relationships with others [15, 25]. Therefore, the influence exerted by family and friends is very important in our lives since it can directly affect decision-making and condition attitudes and behaviour [41].

Several studies have shown that enjoying a vacation with family or friends is beneficial for social relationships [16]. In addition, vacations help create close bonds that increase sociability [29], promote face-to-face communication [45], build trust [26] and generate social and family commitment [39].

Jiang and Balaji’s (2021) research identifies several reasons for tourists to participate in a DFT trip, including social and family engagement, connection with nature, relaxation and novelty, which all increase well-being during holidays. It can even reinforce bonding points with loved ones without the constant need to send social media notifications and enhance that active engagement with a DFT experience [7].

Family and social commitment can represent a barrier to holiday enjoyment that must be negotiated and addressed personally by the potential tourist as technology wields extensive power in the experience [28].

### Nature connectedness

Other research suggests that social and family engagement can be enhanced with an immersive experience in the natural environment [7]. Nature connectedness has been defined as the subjective feeling of association with the environment that implies meaningful participation in something greater than oneself and that can be related to scales of natural emotional, social and psychological well-being [46, 47]. Researchers have found that immersion in a natural environment creates positive communication bonds, enhances the development of personal skills, reinforces attachment and interpersonal harmony, and increases sociability [15, 46, 48]. When choosing an experience of well-being and relaxation with family and friends, DFT in nature gives the tourist a chance to try new activities that favour full enjoyment of the environment [26] and strengthen social and family bonds [38]. Nature can enhance self-expression and self-control and contribute to a healthy experience [11].

DFT limits the constant presence of IT with activities in environments that allow tourists to enter the natural environment [49] and engage with family, friends and fellow travellers in activities that improve interpersonal relationships without the need to rely on mobile devices. Thus, bonds of unions are reinforced without the constant obligation to send emails, upload photos to social networks or publish videos on the Internet [38, 40].

Hence, the following hypothesis is proposed for research:

*H1. Social and family engagement positively influences nature connectedness*.

### Health and relaxation

One of the consequences of a world with digital communication without limits is the increase in stress levels [21, 50].

Some studies conclude that DFT is a way for tourists to reduce technostress, which is a subtype of stress that is characterized by a loss of control due to being connected to the Internet with devices such as smartphones, causing frustration, anxiety and an absence of privacy [51].

DFT can allow tourists to escape from their usual work routines and disconnect in the middle of nature with limited use of IT [29, 52]. This increases the feeling of well-being and relaxation [6, 15, 53, 54]. It also improves the participants’ health by avoiding compulsive use of the Internet in daily online activities, such as posting on social networks, instant messaging, sending and receiving emails or watching online videos [15, 21, 38].

Hence. The following research hypothesis is proposed:

*H2. Health relaxation positively influences nature connectedness*.

### Behavioural intention

Behavioural intention is the subjective probability that a person is going to act in some way and have certain behaviour [41]. In the tourism sector, conceptual models have tried to investigate what factors influence the behaviour of a tourist when choosing a type of experience and how these affect the tourist’s intention to book a trip [50].

DFT reduces the negative impact of technology and the Internet during leisure activities and holidays by limiting the use of digital devices that cause distractions and pathologies [14]. An excessive use of technology causes technostress, depression, low self-esteem, anxiety and other new diseases associated with technological addiction, such as nomophobia, FOMO disorder, and phubbing [55, 56].

This study is based in other research works, but this model has a lot of new apports.

Similar model as [7]. These authors present a model Digital-Free tourism holiday as a new approach for tourism well-being.

Additionally, Previous studies such as Zhuang et al. [57] and Jiang and Balaji [15] have showed different models and relations with some variables, as the positive relation between ‘Use digital technologies during holidays’ in ‘Tourist self-control during holidays’. Egger et al. [32] and Dickinson et al. [26] presented the negative influence of ‘Use digital technologies during holidays’ in ‘Technology dark traits in holidays’.

On the hand, Technology dark traits in holidays’ have a positive influence in DFT [58]. Finally, Jackson [59] and Fong et al. [60] established the influence of ‘Tourist attribution’ in DFT.

Several investigations have concluded that certain factors, such as social and family engagement, nature connectedness and health relaxation, favour the intention to participate in a DFT experience and positively affect tourists’ behavioural intention [7, 15, 29].

Social and family engagement can influence tourists’ intention to choose a DFT experience, and an increasing number of friends, family and private circles recommend enjoying DFT trips [49].

Due to the above literature, the following research hypothesis was proposed:

*H3. Social and family engagement positively influences behavioural intention*.

As seen above, an immersive trip in nature can motivate a person to escape from a hyperconnected world [18, 26]. This increases tourists’ enjoyment of the trip [29]. This approach has been supported by other studies researching digital disconnection experiences at destinations surrounded by nature, such as campsites [26], detox retreats [61] or mountain huts [49]. All of these factors provoke positive and authentic emotions in tourists who consider them decisive elements when making a DFT trip with full immersion in nature [6, 62].

The contributions to well-being and health of this type of trip means that Behavioural Intention is positive when connecting with nature on a DFT experience [7, 15, 63].

Hence, the following research hypothesis is proposed:

*H4. Nature connectedness influences Behavioural intention*.

In addition to social and family engagement and nature connectedness, the desire for relaxation and health is also an element that can condition the decision to choose a DFT destination [20, 50]. Numerous studies address the negative impacts of technology addiction and its harmful effects on health [64]. DFT has a high demand from users who want to mitigate the negative effects of hyperconnection and find enjoyment, pleasure and spirituality [7, 26, 29]. Suppliers in the tourism sector have tried to channel this intention to meet the demand for the well-being of their customers [18, 35, 65].

The following research hypothesis is proposed using the above:

*H5. Health relaxation positively influences behavioural intention*.

### Economic sustainability and sustainable tourism

The revolution and transformation of tourism caused by IT plays a fundamental role in world economies [7]. In 2030, the United Nations World Tourism Organization program predicts that there will be over 1,800 million tourists [1]. This will generate income, create new jobs and promote economic opportunities that can increase the sustainability and profitability of the tourism industry [66].

Technical, social, environmental, economic and political challenges all affect demand and sustainability in many countries that already promote tourism in nature [67]. The economic sustainability of tourism should allow for viable economic projects in the long term, which produce socioeconomic benefits for all stakeholders. These include alleviating poverty, income-generating opportunities, stable employment, and social services for host communities [1]. Therefore, sustainability must satisfy the different stakeholders so that there are positive feelings in social commitments, defence of natural resources and improvements of the tourist experience [34]. DFT can be relevant for the sustainability and profitability of tourist destinations and is important for their economy [4]. In addition, DFT aims to maintain tourist satisfaction and ensure that tourists live a meaningful experience that will make them aware of sustainability issues and sustainable tourism. Existing studies indicate that tourist awareness is being attracted to new sustainable experiences that are completely different from saturated mass tourism and focus on well-being and authenticity at a DFT destination [49, 68]. A DFT tourist seeks a balance between good infrastructure, safety, healthy activities, new experiences, personalized offerings and respect for the environment [9, 17, 36] and an experience that includes quality services that protect nature, ecology and control to reach more efficient, sustainable services without noise or light pollution [7, 54]. All these elements are an integral part of sustainable tourism for economic development, society and the environment [69].

The opportunity that DFT gives for business growth and job creation [35, 70] as a new market niche for companies and new entrepreneurs can have an impact on the decision of the DFT tourist and condition their behavioural intention for a trip [24, 34]. This means that tourist destinations must promote and specialize in these types of experiences [7, 16, 50].

The last research hypothesis is proposed based on these studies:

*H6. Behavioural intention positively influences economic sustainability*.

**Fig. 1:**
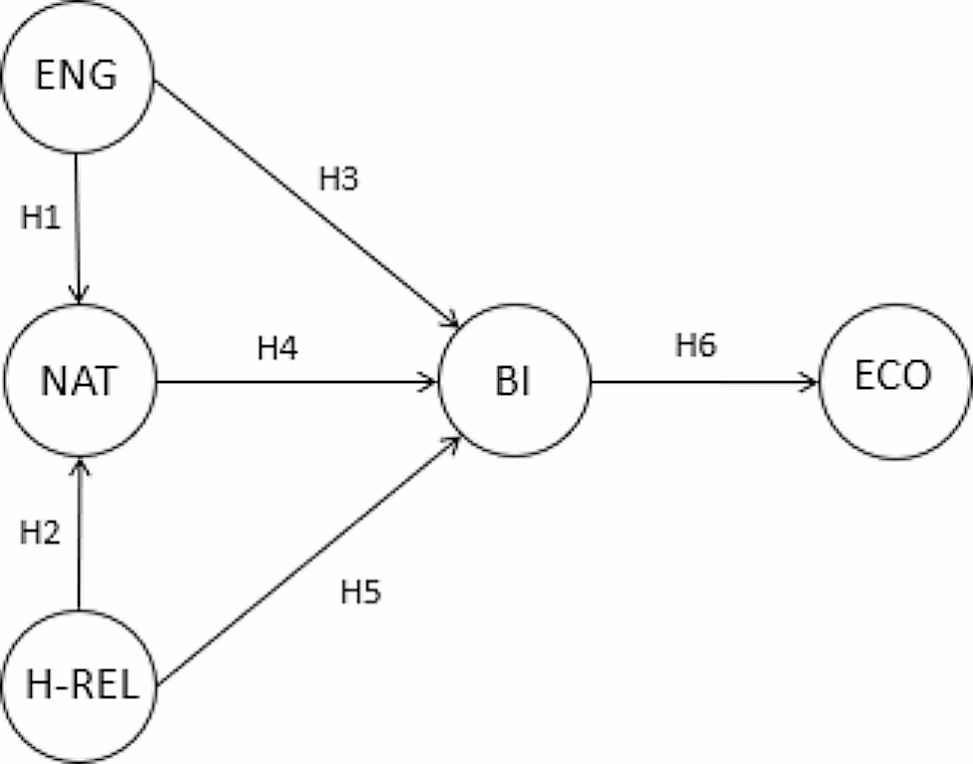
Theoretical model. *Note*: ENG (Social and Family Engagement), NAT (Nature Connectedness), H-REL (Health-Relaxation, BI (Behavioural Intention), ECO (Economic sustainability). *Source*: Authors

The relationships between the distinct factors are shown in Fig. 1.

## Methods

The objective of this research is to advance the knowledge of new structures of motivational factors that can understand the decision of a tourist to make a DFT trip. To this end, it is investigated whether family and social engagement and health and relaxation have a positive impact on the behavioral intention of the potential tourist and whether this influences sustainability due to the importance of DFT in the new economic framework.

For this purpose, a quantitative approach has been used with an online survey including question areas from previous studies [7, 15, 24, 29].

The questionnaire investigates the profile, attitudes and motivations of DFT tourists [4, 71, 72]. This allows tourism service providers and managers to consult this research and adapt marketing strategies to tourists who demand these types of wellness and health services.

### Data collection

The answers to questions about the proposed relationships and the influence of each dimension of sustainability of DFT were measured with a five-point Likert scale (5 = “strongly agree”, 1 = “strongly disagree”) [73]. The study uses a conceptual model that analyses the interrelationships of the variables that contribute to behavioural intention for the DFT experience.

The methodology employed is a questionnaire in an attempt to reach a broad audience. In our research, we conceptualise sustainability DFT as a pioneering study through an analysis of PLS-SEM results that can contribute to critical debates in technology and tourism studies. The common method of bias with the Harm test has been taken into account [43]. The model is used to analyse the influence of the above variables on economic sustainability and sustainable tourism.

The theoretical model in the proposal above (see Fig. 1) connects social and family engagement, nature connectedness and health-relaxation variables to behavioural intention for DFT and the contribution to economic sustainability.

The indicators selected in previous studies were also analysed. The most important studies and elements in the literature were reviewed [7, 15, 24, 29]. To measure sustainability, the scales proposed in previous work were adapted [4, 24, 34], such as DFT experiences generate profitability for the tourism sector, DFT is a driver of future economic sustainability, DFT promotes new jobs and DFT creates new companies and entrepreneurs.

This study uses the proposal of [38] to evaluate health relaxation.

For nature connectedness, the items were proposed using the work of [7, 15, 26]. Social and family engagement items were adapted from those used by [11, 16, 38]. The analysis of behavioural intention was based on previous work by [7, 15, 29, 49].

The items included for each construct are shown below in Tables 1 and 2. The measurement scales that were developed and adapted using the literature on previous research are also shown in Table 2.

### Sampling procedure

A specially created online questionnaire was used in the research, and respondents were asked to answer questions about DFT. It is important to note that the questionnaires were anonymous.

The questionnaire has been previously validated with experts from the tourism sector and academics using a Google Forms format. Of the experts, 5 are academics from the University of Extremadura, 2 are researchers from the Lisbon Research Centre and 5 are professionals from the Spanish tourism sector.

The procedure was to use no probabilistic convenience sampling. The questionnaire about tourist destinations, entrepreneurship, mindfulness, relaxation and meditation was advertised on social networks in Spain with the corresponding permission and rights of the respondent.

Stratified sampling by age group was used in training sessions for businesspeople, academics and entrepreneurs, as well as public administration staff and industry professionals who were given the questionnaire for research purposes and collaboration with the study.

The data were collected between July and October 2022 and were first analysed for missing values. Of the 435 questionnaires received, 9 were eliminated due to incomplete or unanswered items and did not count towards the total sample. In the end, 426 questionnaires were obtained with valid responses.

In the questionnaire’s preparation, wording, order and characteristics, it is possible to indicate that [74] recommendations have been taken into account.

In particular, it should be noted that a control question was included to eliminate questionnaires that did not pass this question. Likewise, an item was added to control the error, which turned out to be lower than indicated by these authors.

### Statistical analysis

The statistical programs SPSS and Smart PLS 4 were used to analyse the results [42]. All questionnaire variables were pre-coded.

IBM SPSS Statistics 26.0 statistics software was used to evaluate the data obtained by descriptive analysis using Smart PLS 4 software to confirm the relationships in the model and the research hypotheses [75]. PLS is the most efficient way to analyse data using the SEM methodology since it provides the theoretical and empirical conditions of behavioural and social science and is especially applicable when the conditions for a closed system are not met [76].

PLS was chosen for several reasons: first, PLS imposes no requirement of normality on the data and is a suitable technique for predicting dependent variables in small samples, given a certain degree of quality in the model [77]. Furthermore, PLS is more appropriate when the objective is to predict and investigate relatively new phenomena [78] as is the case of DFT and technology in the tourism sector also applied to business management research [66, 76].

## Results

### Analysis of the measurement model

The reliability and validity of the proposed model are checked to verify that the observed variables accurately measure the theoretical concepts. All the constructs are reflective, which means that the model uses data that have item reliability, with all factorial loads greater than 0.505 [79], presenting values between 0.759 and 0.949. Bootstrapping with significant loads (99.99%) was used to find the t statistics.

**Table 2:** Construct variable measurements: average variance extracted (AVE), composite reliability, Cronbach’s alpha and loadings

Construct	Items	Loadings	Cronbach’s alpha	Composite reliability	AVE
BI-behavioural intention	[ECO1 I would recommend DFT destinations]	0.949***	0.876	0.942	0.890
	[ECO2 I would repeat a DFT experience]	0.937***			
ECO-economic sustainability	[ECO3 DFT experiences are profitable for the tourism sector]	0.812***	0.879	0.917	0.733
	[ECO DFT is a driver of future economic sustainability	0.854***			
	[ECO5 DFT promotes new jobs in the region]	0.888***			
	[ECO6 DFT creates new companies and entrepreneurs]	0.870***			
ENG-social and family engagement	[ENG1 Being offline benefits my social relationships]	0.833***	0.869	0.911	0.718
	[ENG2 When I disconnect, I spend more time with my family]	0.894***			
	[ENG3 Disconnecting favours face-to-face relationships]	0.808***			
	[ENG5, I enjoy the local culture when I disconnect on my trip]	0.853***			
NAT-nature connectedness	[NAT1 I am more at one with nature when I disconnect]	0.895***	0.894	0.934	0.825
	[NAT2 Disconnecting allows me to fully enjoy nature]	0.916***			
	[NAT3 When I disconnect, I feel good in nature]	0.913***			
H-REL-health-relaxation	[REL, I relax when I am offline]	0.865***	0.920	0.938	0.718
	[REL2 When I disconnect, I enjoy things]	0.877***			
	[REL3 Being disconnected gives me peace and well-being]	0.890***			
	[REL4, I feel mindfulness when I disconnect]	0.783***			
	[REL5 Being disconnected allows me to enjoy pleasant sensations]	0.897***			
	[REL6 I am open-minded when I am offline]	0.759***			

The calculations for Cronbach’s alpha for each of the constructs gave values higher than 0.7, which is the established minimum [80]. These values were between 0.869 (Social and family engagement) and 0.920 (Health-Relaxation).

The composite reliability was seen to be internally consistent because all the constructs had values greater than 0.9, which are higher than the proposed minimum of 0.7 (Hair et al. 2011). The results for the average variance extracted (AVE) resulted in values between 0.718 (social and family engagement) and 0.890 (behavioural intention), which verify convergent validity, as they are all greater than the minimum of 0.50 (Fornell & Larcker, 1981) (see Table 2). The calculation of AVE ≥ 0.5 means that more than half the variance of each indicator is explained by the construct [42, 81, 82].

**Table 3:** Discriminant validity

Discriminant validity	Fornell-Larcker					HTMT criterion			
Construct	BI	ECO	ENG	NAT	H-REL	BI	ECO	ENG	NAT-
BI-behavioural intention	0.943								
ECO-economic sustainability	0.798	0.856				0.904			
ENG-social and family engagement	0.526	0.460	0.848			0.595	0.520		
NAT-nature connectedness	0.516	0.449	0.748	0.908		0.580	0.502	0.845	
H-REL-health-relaxation	0.597	0.548	0.813	0.745	0.847	0.664	0.608	0.908	0.819

Table 3 shows how all the indicators used in the research meet the requirements established for discriminant validity, since the diagonal values are all higher than the other values in the same columns and rows [79].

In addition, the heterotrait-monotrait criterion (HTMT) was calculated to find the discriminant validity. The values of HTMT must be less than 1 to show discrimination of two factors [77]. Table 3 (final columns) shows that all variables had discriminant validity when following the criteria for HTMT.

From the results obtained, the measurement model was considered to have sufficient levels of validity and reliability, and the evaluation of the structural model can proceed.

### Structural model analysis

Once the measurement model validity has been verified, the structural model of the different constructs is analysed to evaluate the coefficient and path significance [80]. The values of R^2^, which is the explained variance of the latent dependent variables, verify that the endogenous constructs of the model are predictive and explanatory [83] (see Table 4).

**Table 4:** Endogenous variables

Hypothesis/construct	R^2^	Direct effect (β)	Correlation	Explained variance
H1(+). ENG *>* NAT		0.798	0.748	31.21%
H2(+). REL *>* NAT		0.061	0.745	30.29%
***NAT***	0.615			61.5%
H3(+). ENG *>* BI		0.421	0.526	3.33%
H4(+). NAT *>* BI		0.140	0.516	9.67%
H5(+). REL *>* BI		0.443	0.597	23.90%
***BI***	0.369			36.9%
H6(+). BI > ECO		0.403	0.798	63.8%
***ECO***	0.638			63.8%

The model explains 61.5% of nature connectedness, 36.9% of behavioural intention and 63.8% of economic sustainability.

Student’s two-tailed t-distribution was used to compare the significance of β coefficients using a bootstrapping process with 5000 samples [80]. The values for the constructs of the model (standardized β path coefficients) are greater than 0.2 [84] or have t values greater than 1.96, apart from the relationship between social and family engagement and behavioural intention.

This means that all the proposed hypotheses used in the structural model were significant except for the hypothesis about the relationship of social and family engagement and Behavioural Intention because this does not reach the minimum accepted value for the t statistic (see Table 5).

**Table 5:** Hypothesis support

Hypothesis	β	T-value bootstrap	P values	Support
H1. Social and family engagement -> Nature connectedness	0.421***	7.682	0.000	Yes
H2. Health-Relaxation -> Nature connectedness	0.403***	7.310	0.000	Yes
H3. Social and family engagement -> Behavioural intention	0.061***	0.805	0.421	No
H4. Nature connectedness -> Behavioural intention	0.140***	2.397	0.017	Yes
H5. Health-Relaxation -> Behavioural intention	0.443***	5.925	0.000	Yes
H6. Behavioural intention -> Economic sustainability	0.798***	37.876	0.000	Yes

*Note*: Bootstrapping 95% confidence interval using 5000 samples

*p value < 0.05, using t (4999), one-tailed test, **p value < 0.01, using t (4999), one-tailed test and ***p value < 0.001, using t (4999), one-tailed test

*Source*: Author

Similarly, the p-values are also less than 0.05 level of significance, except for H3 which is the positive influence of Social and family engagement on Behavioural intention. The value obtained is higher (0.41) and is not supported because the significance level is higher than the 0.05 threshold, which means that the confidence level is lower than 95%.

## Discussion

The first and second hypotheses are validated, which show that both ENG and REL have a positive influence on BI and NAT [27, 32, 41]. This coincides with the findings of [29, 49] and therefore validates the research hypothesis.

Other authors, however, consider that the constant need for commitment to the family is an obstacle to enjoyment and creates an obligation to communicate. This can cause frustration and discomfort and means that tourists are under pressure because they do not have the necessary language skills to communicate [71, 78]. This negative feeling is highest in a natural, isolated and unconnected environment [50, 85]. proposes that this drawback does not influence behavioural intention for connection with nature with DFT. Being in a cabin in the forest can help visitors gain self-knowledge and immerse themselves in the environment, but this does not happen in places such as hotels or urban resorts where the feeling of being in a natural environment can be blurred and therefore reduce the enjoyment of the natural environment.

On the other hand, social commitment, defined as the process of establishing and improving ties with family and friends, has a positive influence on tourist motivation to participate in a DFT trip and therefore has an influence on tourist intention to experience DFT [7, 29, 72].

However, contrary to what is proposed in the third research hypothesis, ENG does not positively influence BI. Some authors affirm that it is not a predictor of DFT intention [50] because social bonding does not necessarily occur due to DFT experience but is gained from different activities that tourists do together in the company of others while on holiday. This may be because our family and friends are connected to the Internet and social media, and the best way to connect with them is digitally; thus, in these circumstances, being disconnected does not benefit social relationships [15, 48].

On the other hand, the results suggest that nature connectedness and health relaxation contribute positively to behavioural intention, especially the first construct, so the fourth and fifth hypotheses are validated.

These results are consistent with the idea that factors of health relaxation and nature connectivity during a trip are decisive when recommending or repeating a DFT trip. The feeling of unity with the natural environment is an attractive reason for a DFT experience is an idea proposed in the scientific literature [6, 7].

On the other hand, relaxation influences motivation to make a DFT trip. A better sensory experience, feeling of freedom, sensory experience and relaxation are possible rewards after engaging in activities without digital media [39]. Relaxation means feeling peaceful and quiet while refreshing the body and mind, which is in line with studies that have found that relaxation can motivate tourists to go sightseeing without digital devices [32, 49]. The results of these studies try to explain that tourist intention to not use digital devices during their holidays has its origin in the belief that a DFT trip will allow a person to feel relaxed and mindful, allow them to express themselves and help them avoid technostress [29, 68]. Other studies also support this theory about the benefits of DFT for improving health and well-being and increasing relaxation and satisfaction [6, 26, 38]. In addition, relaxation and mindfulness have positive impacts on tourist intention to travel by limiting the use of technology [3, 9].

In other lines of research, it is concluded that the excessive use of new technologies minimizes commitment to family and social relationships [29].

The sixth hypothesis predicts that Behavioural Intention positively influences economic sustainability [34, 54]. The results reveal (β = 0.xxx. t = 2. xxxx) that the hypothesis is supported. These data are in line with the studies of [24, 40, 70].

**Fig. 2:**
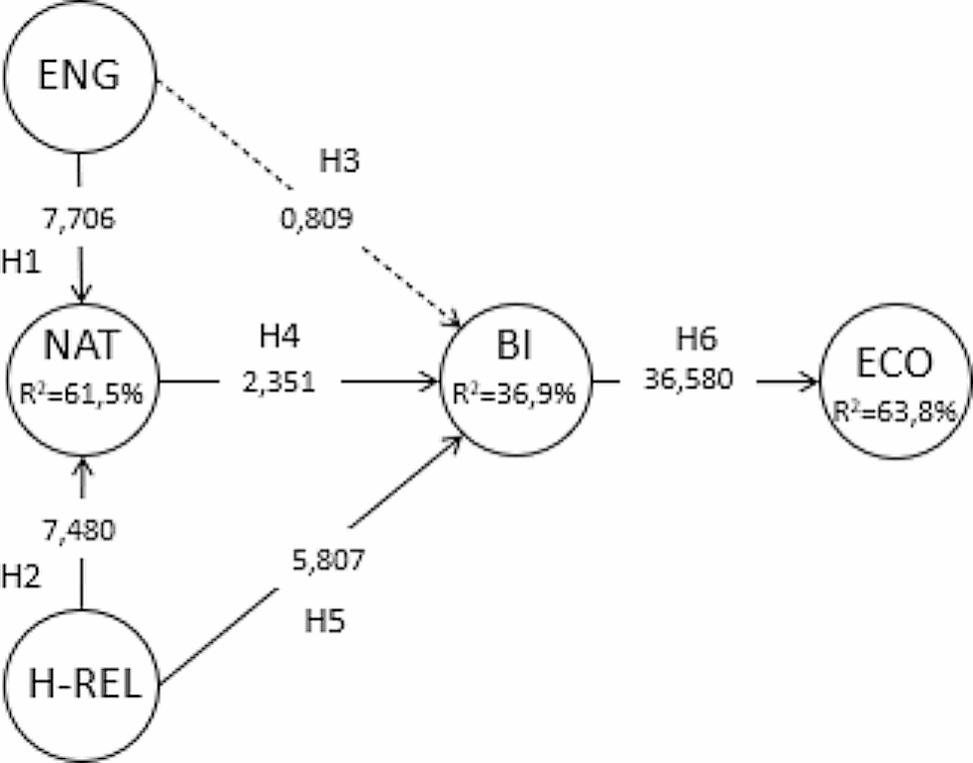
Graph of the structural model analysis results. *Note*: ENG (Social and Family Engagement). NAT (Nature Connectedness). H-REL (Health-Relaxation). BI (Behavioral Intention). ECO (Sustainable Economy). *Source*: Authors

Figure 2 presents the model with the confirmed relationships of the research, the trajectory results and their statistical significance.

The above data justify proposing a new tourism product based on the voluntary absence of technology during a trip [7, 16] to promote the sustainable economy of a territory [4] because behavioural intention clearly influences economic sustainability.

This confirms what most authors in the peer-review literature propose [14, 15] for five of the research hypotheses used in this study. Four different elements of motivation that positively affect behavioural intention to go on a DFT trip have been identified. These are economic sustainability, social and family engagement, nature connectedness and health relaxation.

## Conclusions

### Theoretical implications

Theoretical contribution with DFT as a driver for attracting potential tourists to help service providers to offer efficient, sustainable services to support the health and wellbeing demanded by tourists who wish to digitally disconnect. DFT can be a driver of economic sustainability and health and wellness therapy in tourism in the digital age.

Innovative technologies are increasingly important as a fundamental part of the tourist experience, and this study contributes to the scientific literature on the topic and adds to the limited number of studies on the motivation of tourists to go on a DFT trip. It advances knowledge by proposing a new structure of motivational factors that could explain the decision of a tourist to make a DFT trip.

To this end, it empirically proposes how variables such as social and family commitment, connection with nature, relaxation or preference for economic sustainability influence the decision to make a trip that is free from technology and digital devices. Study participants have consistently indicated the positive impacts that temporary abandonment of digital devices can have during holiday periods.

This empirical study also expands the lines of research on DFT and proposes new dimensions to try to lay theoretical foundations for future studies into DFT, such as disconnection from work, privacy or sustainable tourism and the positive impacts on the decision to choose to disconnect digitally while taking a trip.

The study shows a great variation in the traveller’s desire to disconnect, as some already want to disconnect digitally, while others live attached to their devices and make them an integral part of their lives. Much of the debate about hyper connectedness and the ubiquity of new technologies has focused on data given empirically in this research, concluding that the decision to disconnect from DFT is complex and that it is not just an individual choice but has other factors inhibiting voluntary disconnection that are all influenced mainly by the social environment of work and family.

### Practical implications

Being disconnected while travelling is an added value for DFT tourists. This has obvious advantages and means it can become part of the creation and design of products and DFT service packages with companies in the sector. All this can result in increased productivity and contribution to well-being, sustainability and an improved lifestyle.

At the same time, it offers an opportunity for small and medium-sized companies to turn the disadvantage of lack of technology into a defining advantage for their product. DFT proposes an adequate use of existing resources that can be improved with efficient strategies and does not require large infrastructures and investment.

Therefore, the practical findings of this research are that digital connections alter the travel experience and the evolution of the rapid adoption of recent technologies in tourism. The omnipresence of digital connections is also changing, and a social transition is beginning for the connection-disconnection dilemma in tourism.

### Limitations and future research

DFT is an alternative and emerging trend that companies and the tourism sector can use to adapt offers to changing market needs. Disconnecting from the digital world, for leisure and for treatment, can be used to create a catalogue of services that can generate new jobs and specialize areas, spaces and regions for this type of tourism.

First, this study is limited to only one target audience made up of people of legal age who travel regularly. The complexity of making the decision to disconnect is latent since most of the scientific literature focuses on opinions and not on empirical data. There are individual choices and an age bias that allows us to distinguish digital profiles such as natives, immigrants, generation z, and millennials. There is also only limited empirical research in this area [7, 27, 32, 86].

Second, potential DFT travellers supplied the data collected in this quantitative study. Future research could develop this conceptual model with travellers who have already taken a DFT trip and check the degree of loyalty and recommendations to future tourists, which would allow the factors of intention for these experiences to be researched. A temporary digital disconnection is accepted and considered positive. Encouraging self-awareness, control and moderation at different types of DFT accommodation (resort, hotel, mountain hut, rural accommodation), the various sizes of travel groups (singles, couples, with family, with friends) and a research agenda of travel-related factors can all be used to predict enjoyable elements for DFT travellers and therefore suggest a future roadmap including other conceptual models such as well-being, DFT experience, and loyalty that could all influence decision-making and give a predictive model for DFT traveller services and products.

Third, the conceptual framework can be useful for the future of tourist destinations that promote or specialize in DFT by generating a collaborative ecosystem that would allow for the expansion of the results of other studies, such as creating a network of disconnected tourist destinations or for potential use by addiction treatment centres.

However, some situations are given which help to focus on the study aims. Tourists on a disconnection experience trip may be limited by the potential recall and forgetfulness bias that could be felt in a hypothetical situation and may differ from the way the traveller behaves once disconnected, so this area of research may warrant future lines of research examining how tourists on a DFT experience trip behave.

Studies still must analyse patterns that analyse the intentions of tourists regarding digital disconnection experiences. The relationships between diverse types of DFT in various places around the world can suggest lines and areas of future research whose results can be used by professionals in the tourism sector to make pragmatic efforts to meet the potential DFT demand in the market. The aim is to generate strength and power for remote areas with reduced means of communication and without current tourism development, which can be rural and undeveloped areas away from busy tourist routes and mass tourism destinations. It is also an opportunity for combined destinations to establish a catalogue of innovative DFT services complying with the following characteristics: lack or limited access to IT with leisure activities in an exclusive and healthy environment. This would allow entities to plan strategies and alternatives for tourism development and marketing policies focused on sustainability, relaxation and social and family commitment as valuable elements of well-being when taking part in the experience.

### Electronic supplementary material

Below is the link to the electronic supplementary material.

Supplementary Material 1

Supplementary Material 2

## Data Availability

All data generated or analysed during this study are included in this published article.

## Abbreviations

AI: Artificial Intelligence
BI: Behavioural intentions
DFT: Digital Free Tourism
HTMT: Heterotrait-monotrait criterion
IT: Information Technology
